# THAT'S JUST MY SUPERPOWER: THE ROLE OF NURSING HOME PROVIDER CHAMPIONS IN IMPLEMENTING A PERSON-CENTERED PROJECT

**DOI:** 10.1093/geroni/igac059.442

**Published:** 2022-12-20

**Authors:** Miranda Kunkel, Megan Kelley, Reese Moore, Kamryn Kasler, Chloe Arrasmith, Alexandra Heppner, Kimberly Van Haitsma, Katherine Abbott

**Affiliations:** Miami University, Oxford, Ohio, United States; Miami University, Oxford, Ohio, United States; Miami University, Oxford, Ohio, United States; Miami University, Oxford, Ohio, United States; Miami University, Oxford, Ohio, United States; Miami University, Oxford, Ohio, United States; Pennsylvania State University, University Park, Pennsylvania, United States; Miami University, Oxford, Ohio, United States

## Abstract

Champions are crucial to successful intervention implementation; however, as an area of research champions have only recently begun to gain empirical focus. The purpose of this study was to identify the barriers and facilitators regarding champion qualities using the domain “Characteristics of the Individual” from CFIR. Qualitative interviews were conducted with nursing home provider champions (n=16) who led efforts to create PAL Cards for 15-20 residents and participated in a total of n=50 monthly interviews that were audio-recorded and transcribed verbatim. The following themes regarding characteristics of the individual emerged: champions’ investment in their community, capacity and existing workload, and willingness to ask for help. While some champions saw the value of the PAL Cards, they lacked the bandwidth necessary to widely implement the intervention in their community. Champions who recruited a supportive team and valued person-centered care exhibited success in implementing PAL Cards.

